# Development of Biodegradable and Recyclable FRLM Composites Incorporating Cork Aggregates for Sustainable Construction Practices

**DOI:** 10.3390/ma17215232

**Published:** 2024-10-27

**Authors:** Dora Pugliese, Valerio Alecci, Mohammad Sadegh Tale Masoule, Ali Ghahremaninezhad, Mario De Stefano, Antonio Nanni

**Affiliations:** 1 Department of Architecture, Materials and Structures Division, University of Florence, 50121 Florence, Italy; 2 Department of Civil and Architectural Engineering, University of Miami, Coral Gables, FL 33146, USA

**Keywords:** FRLM composites, lime mortars, cork granules, mechanical properties, hygroscopic performance

## Abstract

Reducing energy consumption in the building sector has driven the search for more sustainable construction methods. This study explores the potential of cork-modified mortars reinforced with basalt fabric, focusing on optimizing both mechanical and hygroscopic properties. Six mortar mixtures were produced using a breathable structural mortar made from pure natural hydraulic lime, incorporating varying percentages (0–3%) of cork granules (*Quercus suber*) as lightweight aggregates. Micro-computed tomography was first used to assess the homogeneity of the mixtures, followed by flow tests to evaluate workability. The mixtures were then tested for water absorption, compressive strength, and adhesion to tuff and clay brick surfaces. Adhesion was measured through pull-off tests, to evaluate internal bonding strength. Additionally, this study examined the relationship between surface roughness and bond strength in FRLM composites, revealing that rougher surfaces significantly improved adhesion to clay and tuff bricks. These findings suggest that cork-reinforced mortars offer promising potential for sustainable construction, achieving improved hygroscopic performance, sufficient mechanical strength, internal bonding, and optimized surface adhesion.

## 1. Introduction

The need for reinforcing existing masonry buildings has become increasingly critical, especially in the context of promoting sustainable and resilient construction practices. Masonry structures, often characterized by their historical and aesthetic significance, require retrofitting solutions that not only enhance structural integrity but also align with environmental sustainability. In recent years, Fabric-Reinforced Lime Matrix (FRLM) composite materials have emerged as a promising solution, offering an eco-friendly alternative to traditional reinforcement methods [1,2].

FRLM is a composite system that incorporates natural hydraulic lime (NHL)-based mortars, combined with fabric reinforcements, typically made of basalt, glass, or carbon fibers. These systems enhance mechanical properties such as tensile strength, durability, and bond behavior with masonry substrates, providing a sustainable means of preserving and strengthening historical structures [3,4]. The significance of FRLM materials lies in their compatibility with historical masonry, as they offer enhanced structural performance while maintaining the original aesthetics and material composition of the buildings they retrofit [5].

The use of natural hydraulic lime as a binder in these composites has distinct advantages. Unlike traditional cement-based mortars, NHL mortars allow masonry walls to “breathe” by facilitating the evaporation of moisture, which is essential for preserving the long-term durability of historical structures [6,7]. When combined with fabric reinforcements, FRLM systems improve the overall mechanical properties, offering a solution for masonry retrofitting that supports both structural resilience and environmental sustainability. These features make FRLM systems ideal for buildings located in seismic regions, where enhanced load-bearing capacity and tensile strength are crucial [8,9].

Over the past decade, research has focused on optimizing the interaction between lime mortars and various types of fabric reinforcements, including basalt, glass, and carbon fibers [10,11]. Among these, basalt fabric stands out due to its excellent mechanical properties, such as high tensile strength, durability, and resistance to corrosion [12,13]. Studies have demonstrated that basalt fabric-reinforced mortars exhibit superior performance under seismic loads, making them particularly suitable for strengthening masonry structures in earthquake-prone regions [14,15]. Furthermore, the lightweight nature of basalt fabric reduces the overall weight added to the structure, a critical factor in retrofitting older buildings [16].

In parallel with the development of fabric reinforcements, researchers have explored the potential of lightweight aggregates to further enhance the properties of lime mortars. Aggregates such as expanded clay, perlite, fly ash, and cork have been investigated for their ability to reduce the density of mortars while improving their hygroscopic and thermal properties [17,18]. Lightweight aggregates are especially beneficial in historical masonry, where adding too much weight could compromise the structural integrity of the building. The combination of lightweight aggregates with natural fiber reinforcements, like Posidonia oceanica, which has been shown to increase flexural strength by 58% and compressive strength by 55% after hydrothermal treatment, could further enhance both mechanical performance and sustainability in heritage building conservation [19].

Among these materials, cork granules have emerged as a particularly promising aggregate due to their unique combination of low density, high thermal insulation capacity, and moisture-regulating properties [20,21].

Our research focuses on granulated cork, sourced specifically from Sardinia, Italy, into a lime-based mortar. Beyond being a renewable material, Quercus suber, or cork oak, contributes to soil preservation, carbon sequestration, and water retention.

Known for its resilience to high temperatures and wildfires [22], *Quercus suber* produces cork with a chemical composition that includes ash (0.7%), total extractives (15.3%), suberin (38.6%), lignin (21.7%), and polysaccharides (18.2%), highlighting its suitability as a sustainable material for eco-friendly construction solutions [23].

Previous studies on cork as an aggregate have focused primarily on cement-based composites, highlighting their potential to improve the thermal and hygroscopic performance of mortars [14,15]. For instance, Karade et al. [16] demonstrated that cork granules can significantly enhance the thermal insulation properties of cement-based materials, though their research did not extend to lime-based systems. Similarly, Barnat-Hunek et al. examined the incorporation of cork into mortar mixtures, emphasizing its potential to reduce thermal conductivity. However, most of these studies concentrated on cement mortars and did not fully address the application of cork in NHL-based mortars.

In the context of lime-based composites, the integration of cork has been less thoroughly explored, despite its potential to improve both mechanical and thermal performance [18]. The research of Panesar et al. [21] and Tedjditi et al. [20] highlighted cork’s benefits in terms of moisture regulation and sustainability, but these studies were limited to cementitious matrices and did not investigate the synergistic effects of cork when combined with fabric reinforcements in lime-based systems. This gap in literature underscores the need for further investigation into the potential of cork-reinforced FRLM composites, particularly in applications that require both mechanical strength and improved thermal insulation.

The present study aims to fill this gap by investigating the integration of granulated cork into a lime-based mortar matrix reinforced with basalt fabric. This combination of materials has not been thoroughly explored in previous research, despite its potential to offer a highly sustainable and efficient solution for retrofitting masonry structures. The novelty of this research lies in the optimization of the cork content in FRLM composites, with the goal of enhancing both the mechanical and hygroscopic properties of the material. By doing so, this study seeks to provide a more comprehensive understanding of how lightweight aggregates, such as cork, can contribute to the development of sustainable construction materials that align with modern environmental standards. Specifically, this research aims to achieve this by optimizing the mechanical performance of FRLM composites through the incorporation of granulated cork and evaluating the hygroscopic properties of the modified material to ensure it meets the requirements for retrofitting applications. To achieve these objectives, a series of experimental tests were conducted, including computerized tomography for microstructural analysis, flow tests to assess workability, capillary water absorption tests, and compression and pull-off adhesion tests on various masonry substrates [24]. Surface roughness parameters were also measured to assess the bond behavior of the FRLM composites with clay and tuff bricks, ensuring the reliability and effectiveness of the material in real-world applications.

This research addresses a significant gap in the current state of the art by evaluating the structural and hygroscopic performance of cork-reinforced FRLM composites. By optimizing the integration of cork into lime-based mortars, this study provides valuable insights into the development of sustainable construction materials that not only enhance the structural performance of masonry buildings but also contribute to their long-term environmental resilience. The results of this research are expected to have significant implications for the field of masonry retrofitting, particularly in regions where seismic resilience and hygroscopic performance are critical considerations.

## 2. Materials and Methods

This study involved selecting a commercial mortar reinforced with basalt fabric as the matrix for an FRLM composite due to its excellent compression and adhesion properties when applied to masonry supports. The mortar selection followed specific criteria:

Natural hydraulic lime (NHL) as the binder with natural, recycled, or recyclable aggregates, meeting the environmental standards of EN ISO 14021:2016/A1:2021 [25].

Compliance with EN 998-1:2016 [24] standards for compressive strength (≥1.50 N/mm^2^), thermal conductivity (≤0.1 W/mK), and water absorption by capillarity (≤ 0.4kg/(m^2^·min½)).

Although Mix 1 (control mortar) met these criteria except for thermal conductivity, this was mitigated by adding low-conductivity material. Granulated cork was chosen as the aggregate due to its sustainability and high hygrometric properties [26,27], based on prior studies showing it outperforms other lightweight aggregates like expanded clay [28], perlite [29], and wood ash [30]. Further research supports the use of cork, highlighting its thermal, acoustic, and antifungal properties, making it an excellent choice for lime-based mortars [31,32].

Six mixtures (Mix 1–Mix 6) were created with varying cork content (0–3% by weight). The raw materials used in these mixtures, along with their respective particle size distributions, are as follows:

- Pure natural hydraulic lime 3.5 (grading size 0–0.6 mm).
- Mineral binder (grading size 0–0.1 mm).
- Siliceous sand (grading size 0.1–1 mm).
- Limestone granules (grading size 0–1.4 mm).
- Fine marble (grading size 0–0.2 mm).
- Cork granules (grading size 0.5–0.8 mm).

Computerized tomography and 3D processing verified the absence of segregation in the mixtures. According to ASTM C1437-20 [33] flow tests were performed to assess workability, followed by capillary water absorption tests [34] (ASTM C1403-22a) and compression tests [35] (ASTM C109/C109M-21) on the absorbed specimens.

In the final phase, according to ASTM C1583/C1583M-20 [36] pull-off adhesion tests were conducted on clay and tuff bricks to evaluate the bond between the FRLM composite, and the masonry supports. Surface roughness parameters were measured using an optical profiler [37] (EN ISO 25178-2:2022).

A graphical abstract has been provided as supplementary material, illustrating the procedure and analysis of this research, summarizing the methodology, and highlighting key findings.

### 2.1. Design and Development of the New Matrix

The cork used for this research is a product obtained from processing waste, characterized by a density equal to 90 kg/m^3^ and a grain size range equal to 0.5 ÷ 0.8 mm.

The selected product Mix 1 is a mortar made from pure natural hydraulic lime, siliceous sand, limestone, and fine marble, that respects the limits imposed by EN ISO 14021:2021 [25] and EN 998-1:2016 [24], except for thermal conductivity values. This choice was made because the addition of cork granules, as reported by various studies in the literature [31,38,39,40], can drastically increase the hygroscopic properties. This property would make Mix 1 a real thermal matrix, ideal for seismic and hygroscopic requalification of existing masonry buildings [4]. Table 1 shows the properties of Mix 1 which are reported in the product technical data sheet.

A further important property to observe is the high compressive strength of Mix 1, declared by the technical data sheet to be greater than 15 N/mm^2^ after 28 days of curing, ten times higher than the established limit of 1.5 N/mm^2^ by EN 998-1:2016 [24]. Compared to the pre-established limits, this high value makes Mix 1 an ideal product for this experimentation, as the addition of the cork granules reduces, but not excessively, the general mechanical properties of the mixture [41,42].

The next step was to determine the percentage by weight of cork in granules within Mix 1, to obtain an ideal mortar for creating the new matrix of the FRLM, without modifying the grain size range, workability, and water absorption. According to research findings, in [32] the percentages of cork added to an NHL-based mortars are calculated on weight. Precisely, the control mortar is prepared, to which 2%, 4%, 6%, and 8% of cork in granules by weight is added, and the mechanical compressive strength is classified as CSII for the control mortar and CSI [24] for the mortar with the 8% cork by weight, demonstrating that the mechanical resistance gradually decreases as the cork increases.

Based on this last work analyzed, the mixtures were prepared adding cork percentages by weight at intervals of 0.5%, 1.0%, 1.5%, 2.0%, and 3.0% to 100 g of Mix 1, as indicated in Table 2. With each addition of cork, the workability of the mixture decreased, until it was decided not to exceed 3.0% of the cork.

Regarding the amount of lime and aggregates in the mixtures, it is essential to clarify that the specific quantities of the components in Mix 1 are not explicitly provided, and in this context, the term “binder” (Table 2) refers to the combination of all the components, excluding cork aggregates.

The uniform water amount utilized is equal to 21.5 mL of water per 100 g of binder (in accordance with the recommendations provided by the datasheets), and as this research represents an initial attempt to explore the effects of cork dosage on the properties of the mortar, it was opted to maintain a constant water quantity to ensure consistency and comparability across the different mixtures. Once the mixtures to be used as matrices were identified, an accurate and detailed experimental campaign was carried out, in which the properties of the six mixtures Mix 1 ÷ Mix 6 were analyzed, in terms of segregation phenomena within the mixtures, workability, evaluation of the water absorption properties, compression strength, and adhesion resistance on masonry support.

The cork content for each mixture is as follows (Table 2):

- Mix 1 (0.0% cork): 0 g of cork was added.
- Mix 2 (0.5% cork): 0.5 g of cork was added to 100 g of Mix 1.
- Mix 3 (1.0% cork): 1 g of cork was added to 100 g of Mix 1.
- Mix 4 (1.5% cork): 1.5 g of cork was added to 100 g of Mix 1.
- Mix 5 (2.0% cork): 2 g of cork was added to 100 g of Mix 1.
- Mix 6 (3.0% cork): 3 g of cork was added to 100 g of Mix 1.

### 2.2. Investigation of Segregation Using Micro-Computed Tomography

To ensure the correct design of the mixture, it is necessary to limit the segregation phenomena, i.e., the potential separation of the different components of the mixture. In the case of Mix 1, with the addition of cork granules, it is necessary to verify that the final mixture has a homogeneous level of distribution of the aggregates. X-ray micro-computed tomography (micro-CT) scanning was performed on all six mixtures to obtain a quantitative evaluation of aggregate homogeneity in the specimen [43]. Micro-CT is a non-destructive and non-invasive diagnostic technique capable of three-dimensionally visualizing the internal structure of the analyzed objects.

The scanning was carried out using the high-resolution Bruker Skyscan 1273 micro-CT scanner. A 1 mm copper filter was used which resulted in a voltage of 125, a current of 300 μA, and an exposure time of 800 ms. The average of 12 projections was obtained at 0.6 degrees each, and the pixel size was kept constant at 30 μm for all mixtures. The projections were then reconstructed into a three-dimensional model with Bruker NRecon software version 1.5. A rectangular core of this model—base of 15 by 15 mm and height of 45 mm—was then analyzed using Bruker CTAn software version 1.7. This was performed to avoid possible boundary conditions visualized by the scanner while scanning the cylindrical specimen and sample stage. At this stage, it was possible to visualize the three-dimensional model of the internal microstructure of the mixtures.

In comparison with previous studies, particularly the work by Travincas et al. [44], which extensively reviewed the use of micro-CT in mortar studies, the present research takes a step forward by applying this technique to novel mixtures incorporating cork. Travincas et al. emphasized the utility of micro-CT in characterizing phenomena such as leaching, carbonation, and mechanical damage, as well as the spatial distribution of pores and aggregates in traditional mortars. However, few studies have applied micro-CT specifically to the evaluation of cork distribution within NHL mortars.

While Travincas et al.’s review highlights the advantages of combining micro-CT with other techniques for a more comprehensive characterization of mortar microstructures, this study demonstrates that micro-CT alone can provide sufficient insight into the spatial distribution of aggregates in sustainable composite materials. Furthermore, our findings suggest a high degree of uniformity in cork distribution, which contrasts with earlier reports of aggregate segregation in traditional mortars. This contributes to the advancement of sustainable material formulations by validating the use of micro-CT for ensuring the consistency of aggregate distribution in innovative mixtures.

### 2.3. Determination of the Flow Properties of the Mixtures

According to ASTM C1437-20 [33], the workability of the mixtures was analyzed by flow test, a method to determine the flow of hydraulic mortars using the apparatus described in ASTM C230/C230M-23 [45].

For each mixture, a layer of 25 mm in thickness was placed in the conical mold and tamped 20 times to ensure the uniform filing of the mold. Once the mold was filled, it was lifted away, the flow table was dropped 25 times in 15 s, and the diameter of the mixtures was measured by a caliper along the three lines scribed on the plate.

This study is unique in that it examines the flow properties of NHL mortars containing cork, as opposed to the cement-based cork mortars investigated in previous research, such as in Liu et al. [46]. Their work explored the effects of cork admixtures on the hygro-thermal characteristics of cement–cork mortars, finding that the incorporation of cork led to significant improvements in thermal and moisture insulation. Similarly, other studies, such as those by Pesaralanka and Khed [47], have focused on the flow and mechanical properties of self-compacting mortars containing graphene oxide, while Mermerdaş et al. [48] examined the impact of artificial lightweight aggregates on geopolymer mortars. These studies primarily investigated cementitious materials, with no comparable research on NHL-based mortars incorporating cork.

### 2.4. Evaluation of the Water Absorption Coefficient

The rate of water absorption test was carried out to evaluate the hygroscopic properties and the capillary water absorption coefficient of the new mixtures, following the methodology of ASTM C1403-22a [34].

Three cube specimens 50 mm^3^ in size were prepared for each mixture, for a total of 18 samples, placed in the moist room 24 h after casting. Once removed from the molds, the samples were stored in moisture-tight plastic bags and left to mature at room temperature. At the age of 28 days, the specimens were placed drying in a ventilated oven at 110 °C and weighed at different intervals, specifically: before being placed in the oven, after 24 h, and after 26 h, when an increment of loss of 0.2% of the previous weighing occurred. Then, the top faces of each cube were marked and measured by a caliper, and the initial weight W0 was recorded. In the next phase, the specimens were placed in appropriate prismatic containers 75 mm high and, once placed on 6 mm thick supports, they were immersed in approximately 10 mm of water. The test samples were removed from the container at different intervals from the time of the first immersion, specifically after 15 min, one hour, four hours, and 24 h. Following each interval, the specimens were carefully dried, then weighed and re-immersed, until the last interval.

Once the test procedure was completed, the water absorption at each time interval A_s_ measured in g/100 cm^2^, was calculated using Equation (1) [34]:

(1) $$A_{s}=10000\times \left(W_{T}-W_{0}\right)/(L_{1}\times L_{2})$$

In which:

- W_T_ is the weight of the specimen at time T [g].
- W_0_ is the initial weight of the specimen [g].
- L_1_ is the average length of the test surface of the specimen cube [mm].
- L_2_ is the average width of the test surface of the specimen cube [mm].

### 2.5. Evaluation of the Compressive Strength of the Mixtures

Compressive strength tests were carried out on the eighteen specimens subjected to the water absorption test (three for each mixture), following ASTM C109/C109M-21 [35] with a load increase equal to 100 N/s, until the specimen breaks.

In comparison to previous studies, the findings of this paper demonstrate enhanced compressive strength performance. Parracha et al. [49] analyzed lightweight thermal insulating mortars, noting a reduction in mechanical properties due to the inclusion of lightweight aggregates such as expanded polystyrene and cork. Similarly, Lakreb et al. [50] observed a decrease in compressive strength, with a reduction of up to 42% when waste cork particles were incorporated to improve sustainability and thermal insulation. In contrast, the mixtures evaluated in this study achieved higher compressive strength, as illustrated in Section 3.4. These findings contribute to addressing gaps in the development of materials that combine both structural reinforcement and enhanced performance for existing masonry buildings.

### 2.6. The Pull-Off Test

The pull-off adhesion tests were conducted following ASTM C1583/C1583M-20 [36] standard, which specifies procedures for evaluating the performance of fabric-reinforced composite materials on masonry substrates. The tests were performed on two types of surfaces: tuff bricks and clay bricks. For each mixture, six pull-off adhesion tests were carried out, leading to a total of thirty-six tests for each type of brick. Specifically, six tuff brick specimens and eighteen clay brick specimens were prepared and reinforced, with the dimensions adhering to ASTM standards [36].

The testing procedure began with manual humidification of the upper brick surfaces. A first matrix layer, with a thickness of 6.5 mm, was applied, followed by the placement of a basalt fabric (a balanced bi-axial mesh with a grid size of 17 mm × 17 mm and an equivalent thickness of 0.032 mm). A second 6.5 mm-thick matrix layer was then applied, with special attention given to ensuring the flatness of the surface for proper adhesion to the steel disks. After preparation, all samples were cured for 28 days in a humid environment at room temperature.

Subsequently, the test areas were engraved using a 50 mm diameter diamond core drill. Steel disks (50 mm in diameter) were glued to the drilled areas with a bi-component epoxy resin adhesive and left to dry for 24 h. The pull-off tests were conducted using an automatic pull-off adhesion tester, which applied a continuous tensile load at a rate of 0.03 MPa/s, with the maximum stress limit set at 3.8 MPa.

Determining the type of failure is crucial for evaluating pull-off adhesion tests, with failure modes assessed based on the ASTM 583/C1583M [36] classification:

(a) Failure in the substrate.
(b) Bond failure at the interface.
(c) Failure in the overlay or repair material.
(d) Bond failure at the epoxy/overlay interface.

The tensile adhesion strength *fh* was evaluated using the following Equation (2):

(2) fh=Ph/A

where

- *Ph* is the tensile load.
- *A* is the area of the test specimen [mm^2^]

## 3. Results and Discussion

This section presents the experimental outcomes from the flow tests, micro-computed tomography analysis, water absorption tests, and mechanical assessments of the novel Fabric-Reinforced Lime Matrix (FRLM) composites. While the results show clear trends regarding the influence of cork granules on the matrix properties, it is crucial to delve deeper into the significance of these findings within the context of existing literature. The addition of cork granules within the matrix impacts flow, density, and mechanical properties, providing insights into the potential of FRLM as an innovative reinforcement material for masonry structures. To enhance the depth of this discussion, we will compare our results with prior studies on cork-reinforced composites and similar materials, emphasizing how our research contributes to addressing current gaps in the field, particularly in achieving a balance between mechanical performance and hygroscopic properties.

### 3.1. Results of the Flow Test

The workability of the mixtures was evaluated using a flow test in accordance with ASTM C1437-20 [33], which assesses the flow of hydraulic mortars using the apparatus specified in ASTM C230/C230M-23 [45].

The novelty of this paper lies in using cork as a sustainable aggregate in natural hydraulic lime (NHL) mortars and its impact on flow behavior and workability. Unlike traditional cement-based or geopolymer systems, cork reduces flowability as its content increases, but this is balanced by the absence of segregation.

Other studies have explored similar challenges: Pesaralanka et al. [47] show that the addition of graphene oxide (GO) in self-compacting concrete (SCC) reduces flowability due to its high surface area, but the use of viscosity modifiers helps maintain the desired slump flow. Similarly, Mermerdaş et al. [48] demonstrate that incorporating artificial lightweight aggregate (A-LWA) in geopolymer mortars enhances flowability by preventing the absorption of the alkaline activator solution, thereby improving workability without segregation. Unlike the GO and A-LWA [49,50], which primarily focus on enhancing flow properties, our study highlights that cork in NHL mortars effectively balances reduced flowability with enhanced stability, offering a sustainable alternative without compromising uniformity.

As shown in Table 3, the results indicate a clear reduction in flow percentage from 94% in the control mixture to 33% in Mix 6 as cork content increases, highlighting cork’s effectiveness in maintaining stability without segregation, even at higher concentrations, and demonstrating its potential as a sustainable aggregate in NHL mortars, unlike traditional cement-based mortars.

### 3.2. X-Ray Micro-Computed Tomography (Micro-CT)

**Figure 1:**
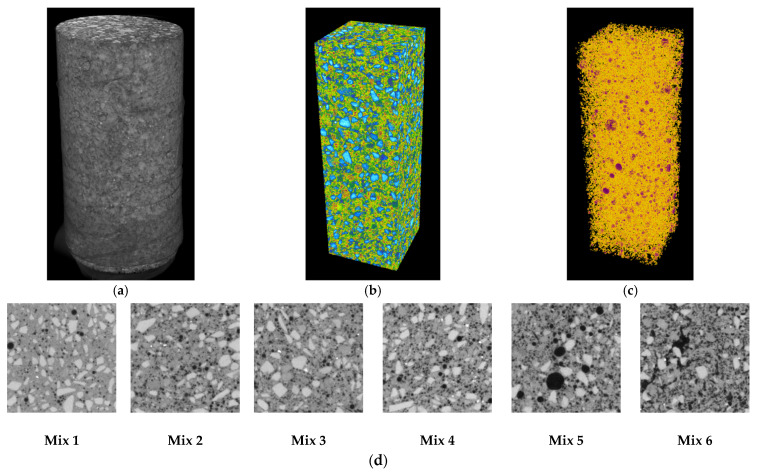
Results of micro-CT scanning and analysis on the samples. Images show (**a**) reconstructed cylindrical sample, (**b**) core sample used for analysis, (**c**) segmented air void and cork volumes, and (**d**) cross-section image of different mixtures.

To acquire the percentage of cork within the mixtures in terms of density and volume, the first step was the segmentation of images using the thresholding technique [51]. Every cross-section was segmented using a grayscale threshold value between 0 (pure black) to 255 (pure white). To keep the parameters constant throughout the analysis, the grayscale threshold 90 was used for all mixtures to represent voids and corks. In Figure 1 the cross-sections of the samples are shown, in which the black color describes very low-density materials, i.e., the air voids and the cork in granules. It is important to understand that after segmentation, the material under study - in this case, voids and corks - are converted to white areas, and the rest of the images are represented as black areas. Therefore, the higher presence of cork correlates with a higher average grayscale index in the cross-section of the segmented image.

**Figure 2:**
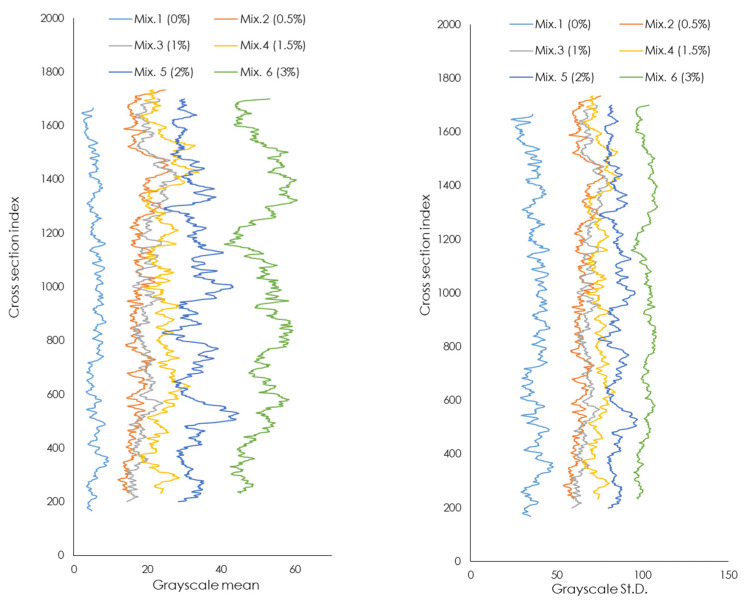
Graphs relating to the analysis of the segmented cross-section images, in terms of (**left**) grayscale mean values, and (**right**) grayscale standard deviation values.

The distribution of the volume of cork granules along the height of each mixture is presented in Figure 2. Graphs relating to the analysis of the segmented cross-section images, in terms of (left) grayscale mean values, and (right) grayscale standard deviation values.

In these graphs, the *y*-axis represents the height of the sample in terms of the number of cross-sections, and the *x*-axis shows the average grayscale value of the segmented image and the relative standard deviation of the values, respectively in the left and right figures.

In the left graph, as the cork granules within the mixture increases, the average grayscale value also increases. The control Mix 1 does not contain any corks and has an almost constant trend, with grayscale values very close to zero. This shows that the distribution of voids is very uniform through the height of the sample. On the contrary, Mix 6 has a very variable trend that oscillates between grayscale values in the range of 40 to 65, displaying that corks do not have a uniform distribution through the height of the sample. The graph on the right presents the results in terms of the standard deviation of the cork distribution along the height, showing a sufficiently stable behavior for Mix 1 and very variable for Mix 6, characterized by a higher standard deviation value than other mixtures.

For the control matrix Mix 1, to which granulated cork was not added, the white color represents the air voids that were generated in the mixture. Knowing the volume quantity of the air voids present in Mix 1, and assuming that the volume of the voids is equivalent in each sample, this value was subtracted from the subsequent volumes, thus obtaining the volume quantity of the cork in granules.

Table 4 shows the results of the three-dimensional analyses. As discussed, the first column shows the volume fraction of void and corks with respect to the entire analyzed volume. The second column shows only the volume fraction of corks in the sample. Third and fourth columns display the total surface area and surface area to volume ratio of the corks. Structure separation is an average value describing the mean distance between each granule with the neighboring cork granules and is a metric of distribution of the corks within the sample.

As the quantity of cork within the mixture increases, the volume fraction of cork gradually rises, until it reaches a volume percentage equal to 18% for Mix 6, i.e., the mixture containing 3% cork by weight compared to Mix 1. The same happens for the external surfaces of the granules: as the cork particles increase, the quantity of the external surface increases. However, it is interesting to observe that the quantities of the ratios between the external surface and the volume of the cork granules decrease as the cork content increases. This phenomenon can be caused by the surface distance between the cork granules, which decreases drastically as the cork increases, appearing barely legible by the scanner, which reads these granules as a single material, and the amount of surface area is consequently reduced.

As the quantity of cork within the mixture increases, the volume fraction of cork gradually rises (Table 5) until it reaches a volume percentage equal to 18% for Mix 6, i.e., the mixture containing 3% cork by weight compared to Mix 1. The same happens for the external surfaces of the granules: as the cork particles increase, the quantity of the external surface increases. However, it is interesting to observe that the quantities of the ratios between the external surface and the volume of the cork granules decrease as the cork content increases. This phenomenon can be caused by the surface distance between the cork granules, which decreases drastically as the cork increases, appearing barely legible by the scanner, which reads these granules as a single material, and the amount of surface area is consequently reduced.

Other research in this field has explored various materials and analytical techniques to understand the internal structure and properties of composite mixtures. For instance, Praneeth et al. [52], while focusing on biochar as a sustainable alternative to sand in cement mortars, make significant contributions by highlighting how biochar affects the porosity of mortars. It effectively demonstrates the potential for biochar to enhance these properties, especially in terms of increasing porosity, which are critical aspects of sustainable construction materials. Similarly, Brunello et al. [53] provide an in-depth analysis of mock-up mortars using a combination of micro-CT and mercury intrusion porosimetry (MIP). This dual approach enriches the understanding of pore size distribution and connectivity within historic and modern mortar compositions. By comparing different analytical techniques, it offers a comprehensive view of how microstructural properties can be assessed, which is crucial for the characterization of building materials, particularly in heritage conservation.

In contrast, our analysis specifically focuses on the precise quantification and segmentation of cork granules within composite mixtures using a consistent grayscale thresholding technique, allowing for an accurate assessment of cork distribution and its effect on structural properties. By maintaining constant parameters throughout the analysis, it provides a more controlled and detailed understanding of how cork influences composite behavior, offering new insights into the potential applications of cork as a sustainable, functional component in building materials. This focused approach on cork granules sets it apart from other research [52,53], contributing to the field with a specialized methodology and a new perspective on material optimization.

### 3.3. Rate of Water Absorption of the Mixtures

To ensure that the classifications of the new matrices comply with those required by EN 998-1:2016 [24], the absorption coefficients by capillarity were calculated, defined as the slope of the curves of capillarity water absorption [kg/(m^2^min^1/2^)]. From the results obtained (Table 6), it was demonstrated that the capillarity coefficient belongs to the Wc1 category (characterized by a capillarity absorption ≤ $0.40\;\mathrm{kg}/(m^{2}\min^{1/2})$) of EN 998-1:2016 [24] only for Mix 3, Mix 4, and Mix 5 mixtures, which presented a capillarity coefficient equal to 0.38 kg/(m^2^min^1/2^) characteristic that makes the analyzed mixtures ideal from a hygroscopic point of view.

Furthermore, it is noted that the control matrix Mix 1 is characterized by a water absorption coefficient equal to 0.57 kg/(m^2^min^1/2^), almost twice as much as stated by the manufacturer’s technical data sheet (Table 6).

Other research has also explored various strategies to enhance the moisture resistance of building materials. For instance, Matias et al. [54] discusses the use of nanomaterials, such as nano-silica or graphene oxide, which can significantly reduce water absorption by refining the pore structure and increasing the density of the mortar matrix. Additionally, the research work also explores the incorporation of natural fibers like hemp or flax, highlighting their ability to improve water resistance while contributing to the mechanical properties and sustainability of mortars. Although effective, these approaches often involve complex preparation processes and higher costs, which may limit their practical application in conventional construction. Liu et al. [46], on the other hand, focuses on using recycled materials like cork to achieve similar outcomes but through different mechanisms, such as altering the pore structure and reducing permeability.

In comparison, our study employs a more straightforward methodology, achieving low water absorption rates through optimized mix design without relying on the advanced additives discussed in [54,55]. The success of Mix 3, Mix 4, and Mix 5 in meeting the stringent requirements of EN 998-1:2016 [24] highlights the potential of conventional materials when used with carefully controlled formulations. This method not only simplifies production but also ensures that the materials remain accessible and cost-effective for widespread use in the construction industry.

### 3.4. Evaluation of Compressive Strength of the Mixtures

For all tests, the average compressive strength value obtained on each specimen is never lower than 1.50 N/mm^2^, a limit established in the first phase of the research, following the specifications of the EN 998-1:2016 [24] standard. As expected, as the percentage of cork present in the mixture increases, the average value of compressive strength progressively decreases, an effect due to the gradual decrease in density. Furthermore, it was observed that the average compressive strength value of control mixture Mix 1 is equal to 5.10 N/mm^2^, three times lower than the value in the technical data sheet, equal to 15 N/mm^2^. Table 7 shows the results obtained.

The average compressive strength f_m_ [N/mm^2^], and the corresponding standard deviation and coefficient of variation.

The average elastic modulus Em evaluated between two points corresponding to 30% and 60% of the maximum stress [56].

Previous research, particularly Tedjditi et al. [55], examined the effect of varying levels of cork absorption on concrete properties, demonstrating that higher cork content leads to a substantial reduction in compressive strength. For instance, when the cork content reached 75%, compressive strength values dropped to between 0.6 N/mm^2^, making the material unsuitable for structural purposes. In contrast, our study shows that even with increased cork content, compressive strength values remain above 1.99 N/mm^2^, meeting the EN 998-1:2016 standard [24]. This demonstrates that the present research has successfully optimized the mixture to maintain mechanical performance while increasing cork content, a balance that previous studies did not achieve.

Lakshani et al. [57] focused on the variability of compressive strength results due to differences in testing methods rather than material composition. While they identified significant discrepancies in strength measurements based on specimen shape and size, they did not explore the impact of incorporating sustainable materials like cork. The current study, by maintaining consistent testing methods and directly evaluating the influence of cork content, provides a clearer and more reliable understanding of how cork affects compressive strength.

Lakreb et al. [50] investigated the partial replacement of cement with cork granules in mortar, finding a 42% decrease in compressive strength with just 2% cork content. The present research advances this understanding by demonstrating that even with a higher cork percentage, the compressive strength remains within acceptable ranges for specific applications.

Overall, the current study makes a significant contribution by showing that cork-reinforced composites can achieve both environmental sustainability and mechanical reliability. By adhering to the EN 998-1:2016 standards [24] and ensuring compressive strength values remain above 1.50 N/mm^2^, the research presents a viable option for sustainable construction, demonstrating innovation and practicality not fully realized in prior studies.

### 3.5. Bond Strength of the Mixtures

**Figure 3:**
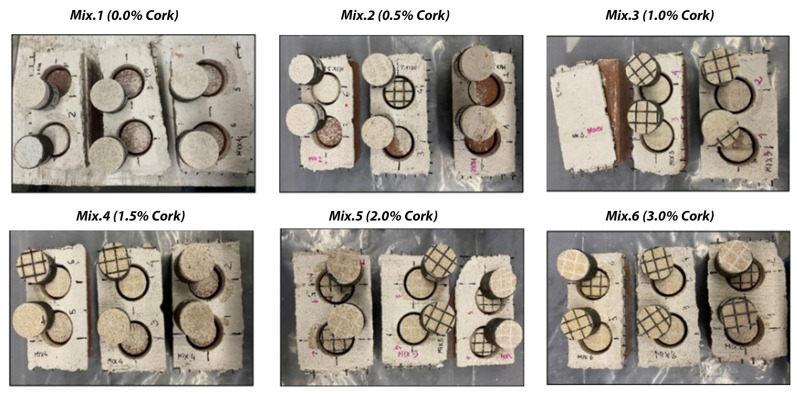
Configuration of the clay bricks after pull-off test (Mix 1–6 L).

Regarding the failure mode, the results obtained from the pull-off tests demonstrated that the six matrices behaved well when applied to tuff bricks. In Figure 3, each mix contains two circular test sections where the pull-off force was applied to the clay bricks. The appearance of damage or separation around the circles indicates varying degrees of failure in adhesion strength, influenced by the percentage of cork added to the mixtures. The bond strength values obtained on the reinforced clay bricks showed considerable variation, primarily due to the different surface roughness of the bricks used.

**Figure 4:**
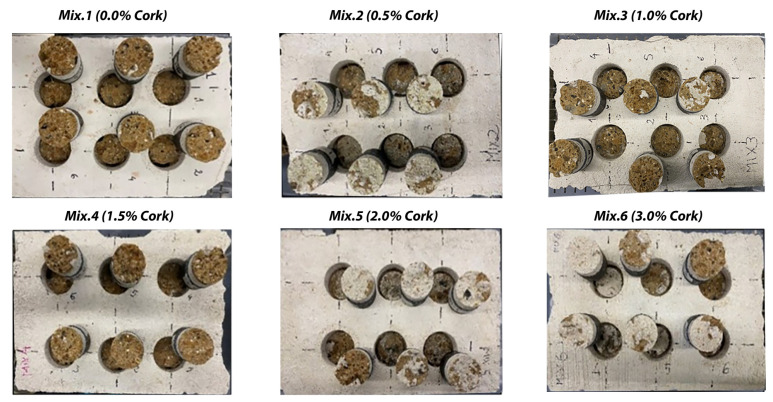
Configuration of the tuff bricks after pull-off test (Mix 1–6 L).

In contrast, Figure 4 illustrates six sets of samples from the pull-off test applied to tuff bricks, showcasing the results after failure occurred. Each set corresponds to different cork content in the mix, ranging from 0.0% in Mix 1 to 3.0% in Mix 6. The matrices adhered more consistently to the tuff bricks, indicating a uniform performance across the mixes.

The two images provided (Figure 3 and Figure 4), each depicting the results of the pull-off tests on samples made from various cork content mixtures, offer important insights into the material performance post-test for both brick types: Figure 3 presents a top view of the samples after the pull-off test for clay bricks, clearly showing the extent of damage and separation at the interface. This visual allows for immediate comparison of how varying cork percentages influence adhesion strength and failure patterns. The damage around the circular sections offers qualitative information regarding the adhesion quality. Figure 4 displays a similar setup for tuff bricks but focuses on the internal material failure post-test. This view provides a better understanding of internal material cohesion and how cork content affects the nature of failure.

**Figure 5:**
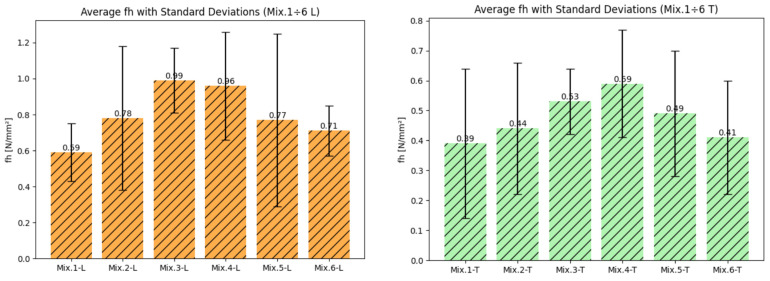
Results of the pull-off test in terms of average f_h_ and standard deviation of clay bricks (Mix 1 ÷ 6 L) and tuff bricks (Mix 1 ÷ 6 T).

Following these observations, the results of the pull-off adhesion tests carried out on both clay bricks (Mix 1–6 L) and tuff bricks (Mix 1–6 T) are presented in vertical bar charts (Figure 5), illustrating the average adhesion strength values [N/mm^2^] along with their corresponding standard deviations. The inclusion of error bars in the bar charts effectively highlights the variations in adhesion performance between the two types of bricks, facilitating a clear comparison of results.

No studies in the current state of the art have explored pull-off tests on tuff and clay bricks reinforced with a composite material incorporating cork as an aggregate in the matrix reinforced with basalt fabric. This research addresses a notable absence in literature, highlighting its innovative contribution to the field and expanding the understanding of FRLM composites and their potential for masonry reinforcement applications.

### 3.6. Evaluation of the Roughness Parameter

The tests carried out on the clay bricks characterized by a smoother surface showed a critical detachment at the matrix-support interface, denoting the complete loss of adhesion. Furthermore, a strong dispersion was observed between the results obtained, depending on the difference in the surface roughness of the brick supports.

In this regard, the analysis of surface roughness was carried out on two types of clay brick surfaces used for the pull-off tests: a smoother one and a rougher one.

To compare the results obtained through the pull-off tests, to be able to state that the FRLM material adheres better to rough surfaces, the surface roughness of the tuff brick was also analyzed.

**Figure 6:**
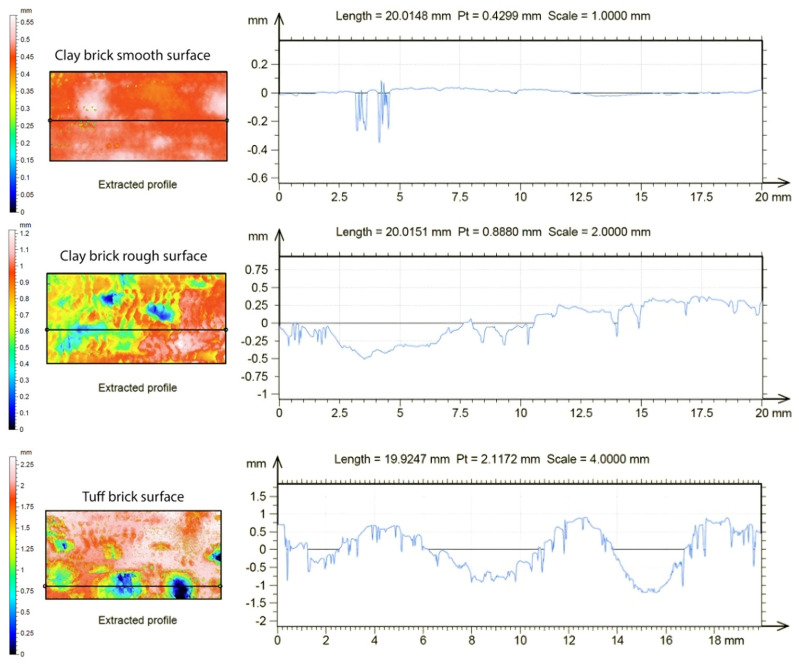
Surface (**left**) and profile (**right**) maps of the smooth clay brick, rough clay brick, and tuff brick.

A non-contact optical profilometer was used to measure surface roughness, in accordance with EN ISO 25178-605 [37]. For the evaluation of surface roughness, the specifications indicated in the European standard EN ISO 25178-2 [37] were followed, where the surface texture parameters and their respective calculation methods are defined (Figure 6). The following parameters were used in this study:

- The arithmetic mean height Sa [mm], which is the mean of the absolute of the ordinate values of the scale-limited surface.
- The kurtosis of the raw profiler Sku, which is the quotient of the mean quadratic value of the ordinate values of the scale-limited surface.
- The skewness of the raw profile Ssk, which is the quotient of the mean cube value of the ordinate values of the scale-limited surface.

In Table 8, it can be observed that clay bricks with a smooth surface are characterized by lower average bond tension values f_h_ compared to those with a rough surface, characterized by a surface roughness Sa equal to 0.163 mm, eight times higher than those with a smooth surface, equal to 0.21 mm. Furthermore, all tests carried out on bricks with a smooth surface always reported adhesion tension values lower than 0.50 N/mm^2^, highlighting how the designed FRLM composite material has better bond behavior when applied on rough surfaces, as observed with the tuff supports, most of which were characterized by a type of failure (a), i.e., by lack of adhesion in the reinforced substrate.

These findings provide a basis for defining a general relationship between surface roughness and the adhesion strength of the applied composite. Specifically, the adhesion resistance tends to decrease when the surface to be strengthened is smooth. While surface roughness has a significant impact on the bond behavior of the FRLM material, it is important to acknowledge that other factors also influence adhesion, such as the mechanical properties of both the matrix and the substrate, as well as environmental conditions. Therefore, surface roughness serves as an indirect but valuable indicator of adhesion potential in this context.

## 4. Conclusions

This study presents a novel approach to developing Fabric-Reinforced Lime Matrix (FRLM) composites by integrating sustainable aggregates, specifically cork granules, to enhance both mechanical and hygroscopic properties, while advancing environmental sustainability. Unlike previous research, this work uniquely combines cork, a lightweight eco-friendly aggregate, with basalt fabric reinforcement in a natural hydraulic lime (NHL) mortar, offering new insights into the balance between performance, sustainability, and practical application.

Several key innovations and technical contributions emerged from this research:

- Flowability: Cork serves as an effective alternative to synthetic additives, stabilizing the mixture without compromising uniformity or workability. Unlike chemical enhancers, cork offers a sustainable solution that aligns with eco-friendly construction practices, reducing the reliance on chemical additives.
- Microstructural analysis using micro-CT: The use of micro-CT scanning to assess the distribution and interaction of cork within the FRLM composite provides detailed insights into the composite’s structural behavior. This methodological innovation enhances the evaluation of lightweight aggregates in construction materials.
- Optimized hygroscopic properties: Cork aggregates improve the hygroscopic performance of the mortar, reducing water absorption without complex additives. This simple, cost-effective method meets EN 998-1:2016 standards [24], demonstrating that sustainability can be achieved with conventional materials while maintaining moisture control.
- Enhanced compressive strength: Despite incorporating cork, the mortar’s compressive strength remains within acceptable standards, challenging the typical trade-off between lightweight aggregates and reduced mechanical strength. This finding underscores that sustainability does not have to come at the cost of performance.
- Surface roughness and bond strength: the research shows that rougher surfaces significantly improve the adhesion of FRLM composites to clay and tuff bricks. While surface roughness plays a key role, other factors, such as the mechanical properties of the matrix and substrate, and environmental conditions, also influence bond strength. Thus, surface roughness serves as an indirect but important indicator of adhesion potential.
- Pull-off strength in FRLM composites: This study presents the first data on the pull-off strength of FRLM composites with cork and basalt fabric reinforcements on tuff and clay bricks, revealing the potential for their application in masonry reinforcement. These findings open new possibilities for FRLM systems in structural retrofitting.

In summary, this research fills critical gaps in the current understanding of FRLM composites by introducing cork as a sustainable aggregate and rigorously testing the material’s mechanical and hygroscopic properties. The novelty of this work lies in its holistic approach to integrating environmental sustainability with structural performance. Further analyses should expand the knowledge gained, providing a solid foundation for practical applications in the seismic and energy retrofit of masonry buildings, with emphasis on durability. Future research should assess the FRLM composite’s long-term performance under various environmental conditions and aging processes. Evaluating the thermal conductivity of the matrices would help assess the composite’s insulation properties compared to existing solutions. Additional mechanical tests, including tensile, shear, and diagonal compression, are needed to confirm its effectiveness for structural applications.

## Figures and Tables

**Table 1:** Properties of Mix 1 are described by the product technical data sheet.

Mix 1. Properties	
Binder	Natural Hydraulic Lime NHL 3.5
Aggregates	Siliceous sand, calcareous, fine marble
Compressive strength σ [N/mm²]	>15
Thermal conductivity λ [W/mK]	0.82
Apparent density of dry mortar [kg/m³]	1580
Adhesion to the substrate [N/mm²]	>1
Water capillarity absorption [Kg/m² min½]	0.3
Grading size [mm]	0 ÷ 1.4
Permeability to water vapor µ [-]	15 ÷ 35
Maximum thickness by layer [mm]	15

**Table 2:** Mixture design of the new mortars Mix 1 ÷ Mix 6.

Mixtures	Binder [g]	Cork in Granules [g]	Mixing Water [mL]
**Mix.1** (0.0% cork)	100	0	21.5
**Mix.2** (0.5% cork)	100	0.5	21.5
**Mix.3** (1.0% cork)	100	1	21.5
**Mix.4** (1.5% cork)	100	1.5	21.5
**Mix.5** (2.0% cork)	100	2	21.5
**Mix.6** (3.0% cork)	100	3	21.5

**Table 3:** Results obtained by the flow test for each mixture.

Mixtures	Initial Diam. [mm]	Diam. 1 [mm]	Diam. 2 [mm]	Diam. 3 [mm]	Average Diam. [mm]	FLOW [%]
**Mix.1** (0.0% cork)	100	194	194	193	194	**94%**
**Mix.2** (0.5% cork)	100	183	184	184	184	**84%**
**Mix.3** (1.0% cork)	100	178	177	177	178	**78%**
**Mix.4** (1.5% cork)	100	142	144	140	142	**42%**
**Mix.5** (2.0% cork)	100	136	136	138	137	**37%**
**Mix.6** (3.0% cork)	100	132	135	133	133	**33%**

**Table 4:** Results obtained from the three-dimensional analysis of the mixtures examined.

Mixtures	Air and Cork Volume Fraction [%]	Cork Volume Fraction [%]	Cork Surface [mm²]	Cork Surface/Volume [1/mm]	Structure Separation [mm]
**Mix.1 (0.0% cork)**	2.2	0.0	0.0	-	1.2
**Mix.2 (0.5% cork)**	6.8	4.6	10,658.4	23.0	0.9
**Mix.3 (1.0% cork)**	7.7	5.4	11,931.3	21.6	0.9
**Mix.4 (1.5% cork)**	9.4	7.2	13,502.6	18.5	0.9
**Mix.5 (2.0% cork)**	12.6	10.4	18,937.3	18.0	0.7
**Mix.6 (3.0% cork)**	20.2	18.0	30,254.6	16.6	0.6

**Table 5:** Comparison between weight percentages and volume percentages of the cork granules within the mixtures.

Mixtures	Weight Percentage of Cork	Volume Percentage of Cork
**Mix.1**	0.0%	0.0%
**Mix.2**	0.5%	4.6%
**Mix.3**	1.0%	5.4%
**Mix.4**	1.5%	7.2%
**Mix.5**	2.0%	10.4%
**Mix.6**	3.0%	18.0%

**Table 6:** Values of capillary absorption coefficient.

Capillarity Water Absorption Coefficient					
Mix.1 (0% Cork)	Mix.2 (0.5% Cork)	Mix.3 (1.0% Cork)	Mix.4 (1.5% Cork)	Mix.5 (2.0% Cork)	Mix.6 (3.0% Cork)
0.57	0.51	0.47	0.38	0.38	0.53

**Table 7:** Result of compression strength, and elastic modulus of the mixtures.

Specimen	Em [N/mm²]	Average f_m_ [N/mm²]	Standard Deviation	Coefficient of Variation
**Mix.1 (0.0% cork)**	431.89	5.10	0.36	0.07
**Mix.2 (0.5 % cork)**	339.64	4.38	0.53	0.12
**Mix.3 (1.0 % cork)**	390.23	4.35	0.05	0.01
**Mix.4 (1.5 % cork)**	433.51	4.03	0.21	0.05
**Mix.5 (2.0 % cork)**	349.75	3.19	0.49	0.16
**Mix.6 (3.0 % cork)**	216.55	1.99	0.12	0.06

**Table 8:** Surface roughness parameters calculated according to EN ISO 25178-2 for bricks with smooth and rough surfaces.

Surface Texture Parameter Defined by UNI EN ISO 25178-2			
	Clay Brick Smooth Surface	Clay Brick Rough Surface	Tuff Brick Surface
S_a_ [mm]	0.02	0.16	0.40
Sku	24.73	3.01	4.25
Ssk	−2.13	−0.62	−1.37

## Data Availability

The raw data supporting the conclusions of this article will be made available by the authors on request.

## Supplementary Materials

The following supporting information can be downloaded at: https://www.mdpi.com/article/10.3390/ma17215232/s1.
