# Supplementary material for: Development of Biodegradable and Recyclable FRLM Composites Incorporating Cork Aggregates for Sustainable Construction Practices
Source: Materials (Basel). 2024 Oct 27;17(21):5232. doi: 10.3390/ma17215232 (PMC11547218; doi:10.3390/ma17215232)
Supplement: Supplementary file 1 [file materials-17-05232-s001.zip › materials-3174610-supplementary.pdf]

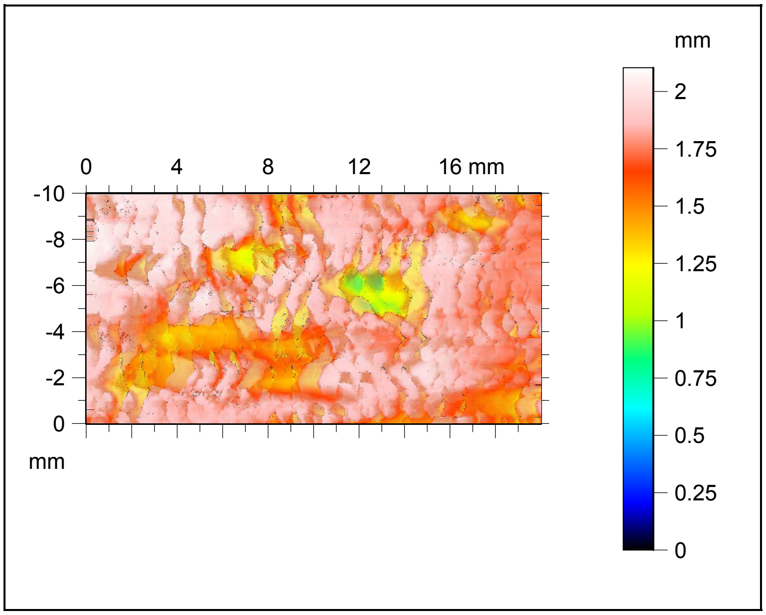

Thresholded Surface

Leveled Surface

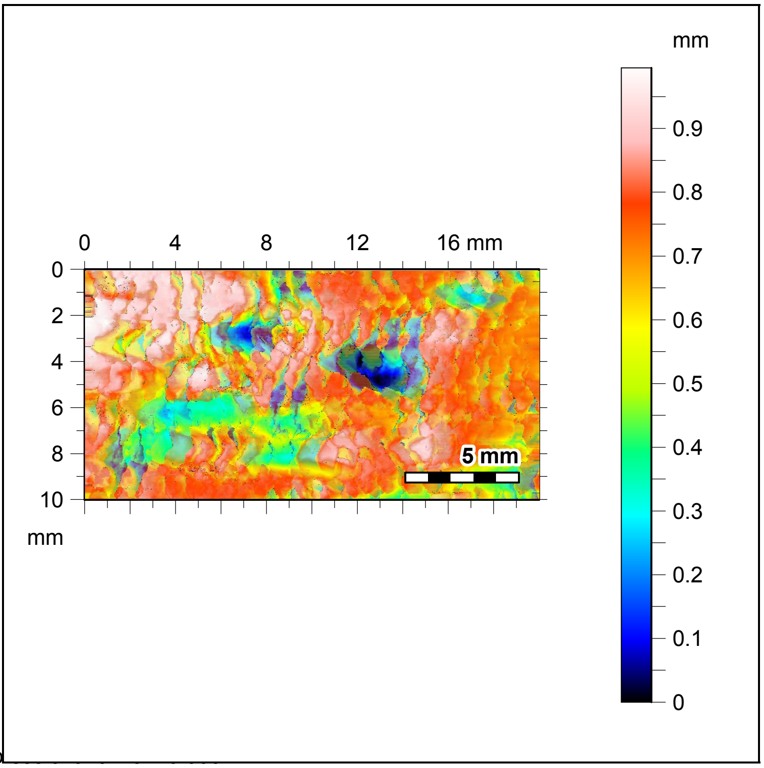

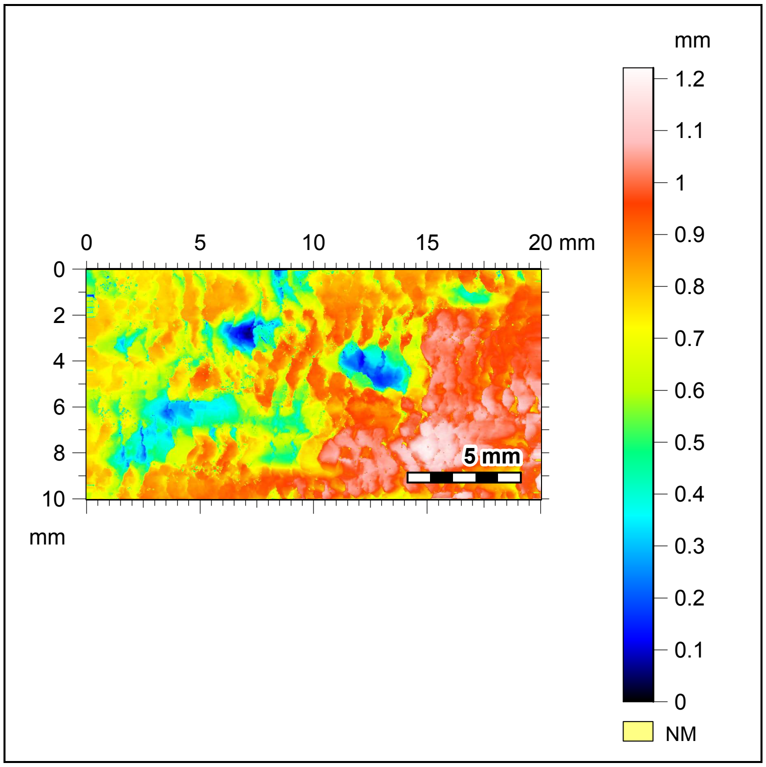

Pro[illegible]

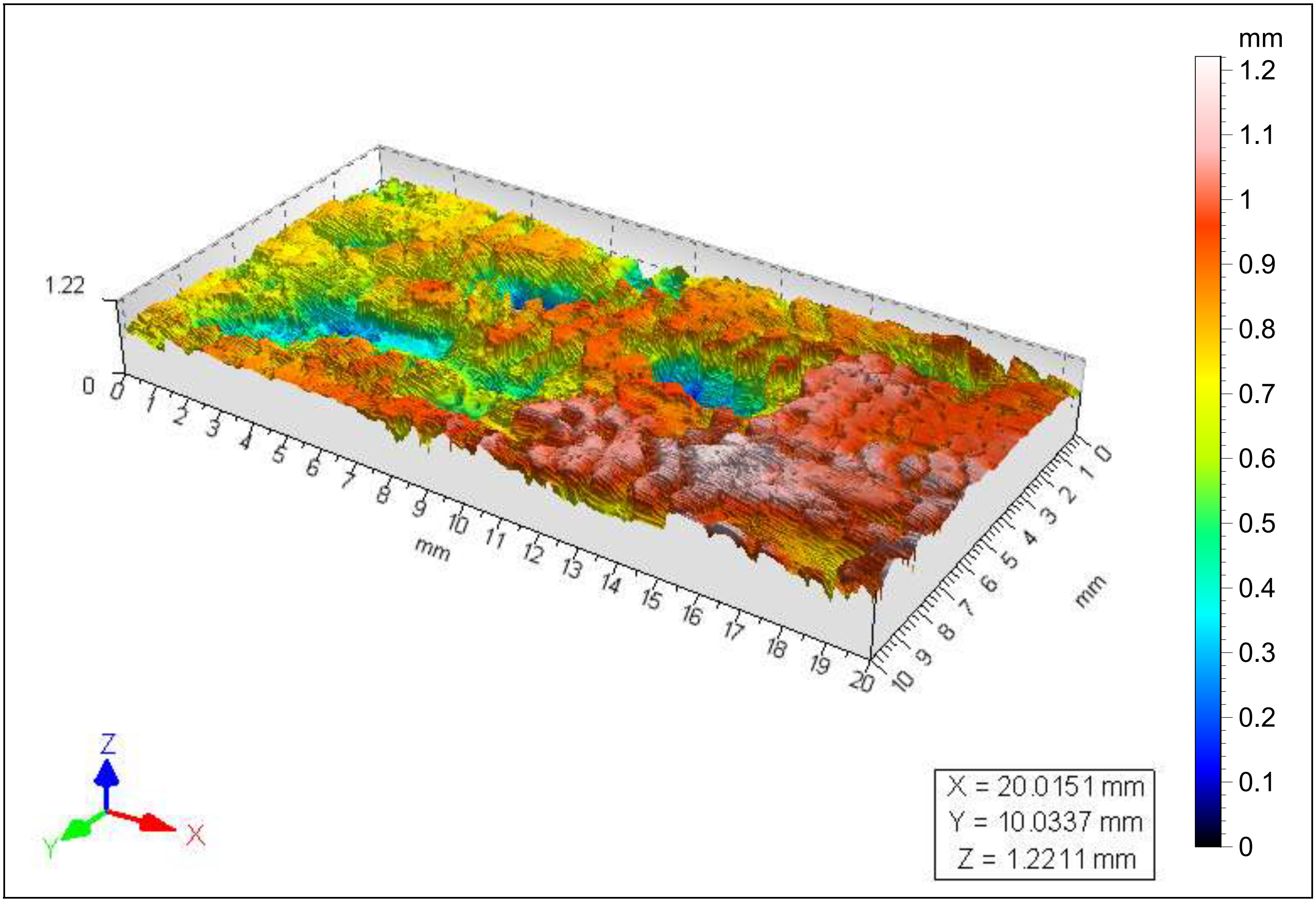

ISO 25178				
Height Parameters				
**Sq**	0.2034	mm		*Root mean square height*
**Ssk**	-0.6242			*Skewness*
**Sku**	3.0135			*Kurtosis*
**Sp**	0.4505	mm		*Maximum peak height*
**Sv**	0.7706	mm		*Maximum pit height*
**Sz**	1.2211	mm		*Maximum height*
**Sa**	0.1629	mm		*Arithmetic mean height*
Functional Parameters				
**Smr**	0.0007	%	*c = 0.001 mm under the highest peak*	*Areal material ratio*
**Smc**	0.2474	mm	*p = 10%*	*Inverse areal material ratio*

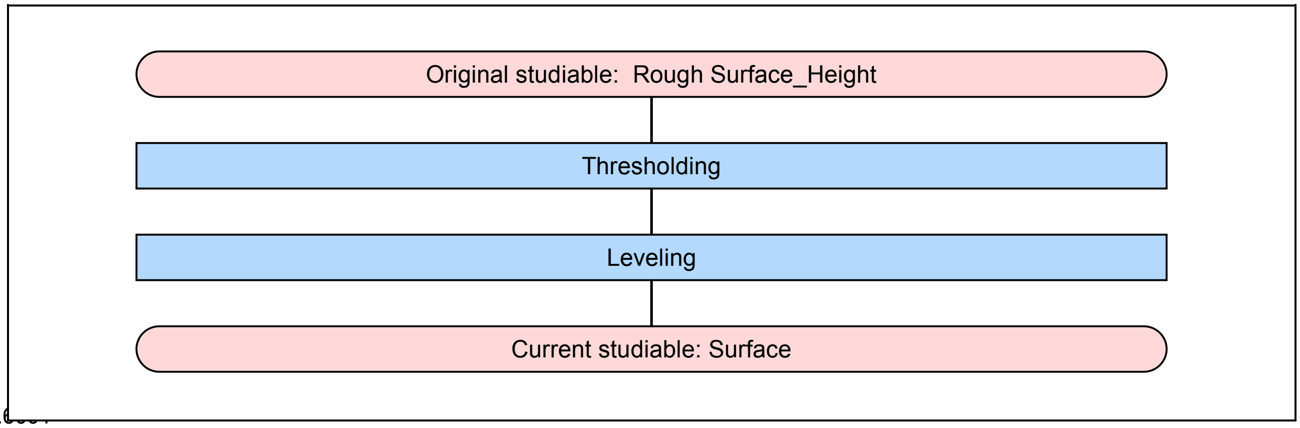

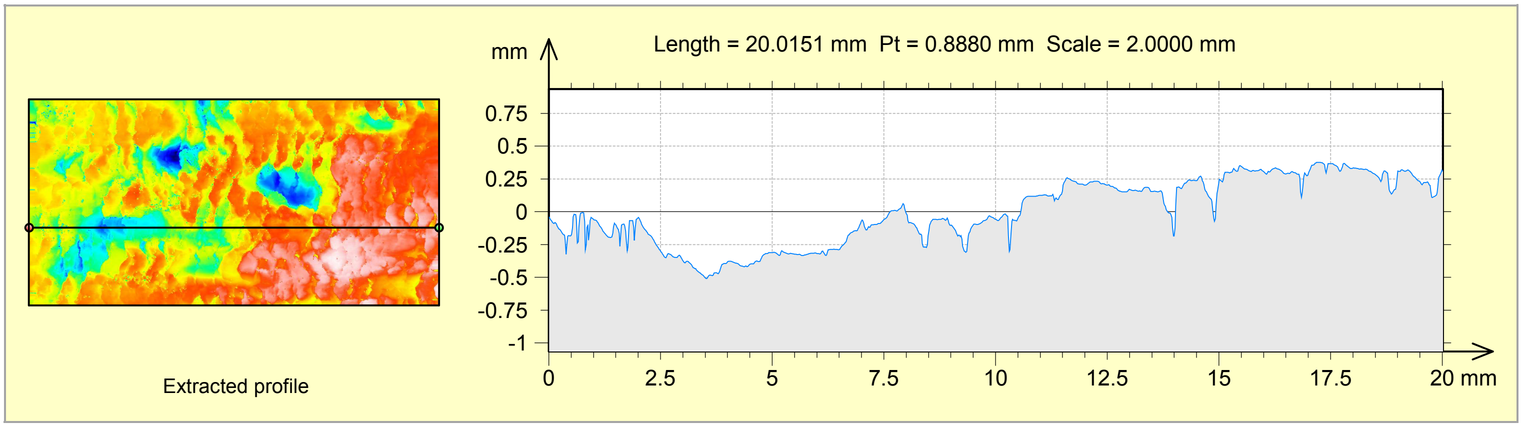

ISO 4287				
**Amplitude parameters - Primary profile**				
**Pp**	0.3790	mm		*Maximum Peak Height of the raw profile.*
**Pv**	0.5090	mm		*Maximum Valley Depth of the raw profile.*
**Pz**	0.8880	mm		*Maximum Height of the raw profile.*
**Pc**	0.4537	mm		*Mean height of the raw profile elements.*
**Pt**	0.8880	mm		*Total Height of raw profile.*
**Pa**	0.2213	mm		*Arithmetic Mean Deviation of the raw profile.*
**Pq**	0.2507	mm		*Root-Mean-Square (RMS) Deviation of the raw profile.*
**Psk**	-0.1958			*Skewness of the raw profile.*
**Pku**	1.7541			*Kurtosis of the raw profile.*
**Spacing parameters - Primary profile**				
**PSm**	2.3720	mm		*Mean Width of the raw profile elements.*
**Pdq**	72.0460	°		*Root-Mean-Square Slope of the raw profile.*
**Material Ratio parameters - Primary profile**				
**Pmr**	0.4610	%	*c = 0.001 mm under the highest peak*	*Relative Material Ratio of the raw profile.*
**Pdc**	0.5540	mm	*p = 20%, q = 80%*	*Raw Profile Section Height difference.*
**Peak parameters - Primary profile**				
**PPc**	0.0000	1/mm	*+/-0.5 mm*	*Peak Count on the raw profile.*
**Amplitude parameters - Roughness profile**				
**Rp**	0.0434	mm	*Gaussian filter, 0.8 mm*	*Maximum Peak Height of the roughness profile.*
**Rv**	0.0736	mm	*Gaussian filter, 0.8 mm*	*Maximum Valley Depth of the roughness profile.*
**Rz**	0.1170	mm	*Gaussian filter, 0.8 mm*	*Maximum Height of roughness profile.*
**Rc**	0.0888	mm	*Gaussian filter, 0.8 mm*	*Mean height of the roughness profile elements.*
**Rt**	0.3177	mm	*Gaussian filter, 0.8 mm*	*Total Height of roughness profile.*
**Ra**	0.0211	mm	*Gaussian filter, 0.8 mm*	*Arithmetic Mean Deviation of the roughness profile.*
**Rq**	0.0288	mm	*Gaussian filter, 0.8 mm*	*Root-Mean-Square (RMS) Deviation of the roughness p*
**Rsk**	-0.4540		*Gaussian filter, 0.8 mm*	*Skewness of the roughness profile.*
**Rku**	4.1475		*Gaussian filter, 0.8 mm*	*Kurtosis of the roughness profile.*
**Spacing parameters - Roughness profile**				
**RSm**	0.4814	mm	*Gaussian filter, 0.8 mm*	*Mean Width of the roughness profile elements.*
**Rdq**	37.8512	°	*Gaussian filter, 0.8 mm*	*Root-Mean-Square Slope of the roughness profile.*
**Material Ratio parameters - Roughness profile**				
**Rmr**	0.1873	%	*c = 0.001 mm under the highest peak, Gaussian filter, 0.8 mm*	*Relative Material Ratio of the roughness profile.*
**Rdc**	0.0310	mm	*p = 20%, q = 80%, Gaussian filter, 0.8 mm*	*Roughness profile Section Height difference*
**Peak parameters - Roughness profile**				
**RPc**	0.0000	1/mm	*+/-0.5 mm, Gaussian filter, 0.8 mm*	*Peak Count on the raw profile.*
**Amplitude parameters - Waviness profile**				
**Wp**	0.0449	mm	*Gaussian filter, 0.8 mm*	*Maximum Peak Height of the waviness profile.*
**Wv**	0.0564	mm	*Gaussian filter, 0.8 mm*	*Maximum Valley Depth of the waviness profile.*
**Wz**	0.1012	mm	*Gaussian filter, 0.8 mm*	*Maximum Height of waviness profile.*
**Wc**	0.4839	mm	*Gaussian filter, 0.8 mm*	*Mean height of the waviness profile elements.*

Pro

**Wt**	0.8383	mm	*Gaussian filter, 0.8 mm*	*Total Height of waviness profile.*
**Wa**	0.2203	mm	*Gaussian filter, 0.8 mm*	*Arithmetic Mean Deviation of the waviness profile.*
**Wq**	0.2246	mm	*Gaussian filter, 0.8 mm*	*Root-Mean-Square (RMS) Deviation of the waviness pr*
**Wsk**	-0.1173		*Gaussian filter, 0.8 mm*	*Skewness of the waviness profile.*
**Wku**	1.2421		*Gaussian filter, 0.8 mm*	*Kurtosis of the waviness profile.*
Spacing parameters - Waviness profile				
**WSm**	2.7924	mm	*Gaussian filter, 0.8 mm*	*Mean Width of the waviness profile elements.*
**Wdq**	10.2656	°	*Gaussian filter, 0.8 mm*	*Root-Mean-Square Slope of the waviness profile.*
Material Ratio parameters - Waviness profile				
**Wmr**	0.6160	%	*c = 0.001 mm under the highest peak, Gaussian filter, 0.8 mm*	*Relative Material Ratio of the waviness profile.*
**Wdc**	0.5509	mm	*p = 20%, q = 80%, Gaussian filter, 0.8 mm*	*waviness profile Section Height difference*
Peak parameters - Waviness profile				
**WPc**	0.0000	1/mm	*+/-0.5 mm, Gaussian filter, 0.8 mm*	*Peak Count on the raw profile.*
**ASME B46.1**				
2D Parameters				
**Rt**	0.3177	mm	*Gaussian filter, 0.8 mm*	*Total Height of roughness profile.*
**Rp**	0.0976	mm	*Gaussian filter, 0.8 mm*	*Maximum Peak Height of the roughness profile.*
**Rv**	0.2201	mm	*Gaussian filter, 0.8 mm*	*Maximum Valley Depth of the roughness profile.*
**Rz**	0.1237	mm	*Gaussian filter, 0.8 mm*	*Average maximum height of the roughness profile*
**Rpm**	0.0519	mm	*Gaussian filter, 0.8 mm*	*Average maximum profile peak height of the roughness*
**Rmax**	0.2284	mm	*Gaussian filter, 0.8 mm*	*Maximum roughness depth of the roughness profile*
**Ra**	0.0210	mm	*Gaussian filter, 0.8 mm*	*Arithmetic Mean Deviation of the roughness profile.*
**Rq**	0.0345	mm	*Gaussian filter, 0.8 mm*	*Root-Mean-Square (RMS) Deviation of the roughness p*
**Rsk**	-1.9866		*Gaussian filter, 0.8 mm*	*Skewness of the roughness profile.*
**Rku**	11.7730		*Gaussian filter, 0.8 mm*	*Kurtosis of the roughness profile.*
**tp**	0.1873	%	*c = 0.001 mm under the highest peak, Gaussian filter, 0.8 mm*	*Profile bearing length ratio*
**Htp**	0.0310	mm	*p = 20%, q = 80%, Gaussian filter, 0.8 mm*	*Bearing ratio height difference*
**Pc**	0.0000	1/mm	*+/-0.5 mm, Gaussian filter, 0.8 mm*	*Peak density of the raw profile*
**Rda**	23.7917	°	*Gaussian filter, 0.8 mm*	*Arithmetic mean slope on the roughness profile.*
**Rdq**	55.2277	°	*Gaussian filter, 0.8 mm*	*Root-Mean-Square Slope of the roughness profile.*
**RSm**	0.5021	mm	*Gaussian filter, 0.8 mm*	*Mean spacing of profile irregularity of the roughness pr*
**Wt**	0.8383	mm	*Gaussian filter, 0.8 mm*	*Total Height of waviness profile.*

**ISO 25178**				
Height Parameters				
**Sq**	0.2034	mm		*Root mean square height*
**Ssk**	-0.6242			*Skewness*
**Sku**	3.0135			*Kurtosis*
**Sp**	0.4505	mm		*Maximum peak height*
**Sv**	0.7706	mm		*Maximum pit height*
**Sz**	1.2211	mm		*Maximum height*
**Sa**	0.1629	mm		*Arithmetic mean height*
Functional Parameters				
**Smr**	0.0007	%	*c = 0.001 mm under the highest peak*	*Areal material ratio*
**Smc**	0.2474	mm	*p = 10%*	*Inverse areal material ratio*
**Sxp**	0.4862	mm	*p = 50%, q = 97.5%*	*Extreme peak height*
**EUR 15178N**				
Amplitude Parameters				
**Sa**	0.1629	mm		*Arithmetic mean deviation*
**Sq**	0.2034	mm		*Root mean square mean deviation*
**Sz**	1.0427	mm		*Ten point height*
**Ssk**	-0.6242			*Skewness*
**Sku**	3.0135			*Kurtosis*
**Sp**	0.4505	mm		*Maximum peak height*

**Sv**	0.7706	mm		*Maximum valley depth*
**St**	1.2211	mm		*Total height*
Area and Volume Parameters				
**Smr**	0.0007	%	*c = 0.001 mm under the highest peak*	*Areal material ratio*
**Sdc**	0.4133	mm	*p = 10%, q = 80%*	*Areal height difference*
**Other 3D Parameters**				
Miscellaneous				
**Smean**	1.7393	mm		*Mean height in absolute*
**Sdar**	*****	mm²		*Developed area*
**Spar**	200.8247	mm²		*Projected area*
**ASME B46.1**				
3D Parameters				
**St**	1.2211	mm		*Maximum height*
**Sp**	0.4505	mm		*Maximum peak height*
**Sv**	0.7706	mm		*Maximum pit height*
**Sq**	0.2034	mm		*Root mean square height*
**Sa**	0.1629	mm		*Arithmetic mean height*
**Ssk**	-0.6242			*Skewness*
**Sku**	3.0135			*Kurtosis*
**SWt**	1.2211	mm	*not filtered*	*Area waviness height*

Identity card

Filename: C:\Documents and Settings\Nanovea\Desktop\Dora\Rough Surface_Height.sur

Axis: X
Length: 19.9980 mm
Size: 1112 points
Spacing: 0.0180 mm

Axis: Y
Length: 10.0080 mm
Size: 557 lines
Spacing: 0.0180 mm

Axis: Height
Length: 1.2658 mm
Spacing: 0.0001 mm

Comments extracted from the studiable:

Measurement Time: 2:47 PM
Measurement Duration: 00:41:07.8750000
Scan Length: 20.0
Scan Step Size: 18.0
Points: 1112

Profile Length: 10.0
Profile Step Size: 18.0
Profiles: 557
Total Points: 619,384

Pen Settings
Pen: 3500
Acqusition Rate: 1000
Averaging: 3
